# Diagnostic Approaches to Carbapenemase Detection in Pseudomonas aeruginosa: A Comparative Review of Phenotypic and Genotypic Methods

**DOI:** 10.7759/cureus.108071

**Published:** 2026-04-30

**Authors:** Bhaskar Y Chavali, Harsha V Patil, Satish R Patil

**Affiliations:** 1 Department of Microbiology, Krishna Institute of Medical Sciences, Krishna Vishwa Vidyapeeth (Deemed to be University), Karad, IND

**Keywords:** antibiotic resistance, blaimp, blandm, carbapenemase-producing pseudomonas aeruginosa, carbapenem resistance genes, carbapenem-resistant pseudomonas aeruginosa, crpa, genotypic detection, phenotypic detection, whole genome sequencing (wgs)

## Abstract

Largely due to the emergence of carbapenem-resistant strains, antibiotic resistance in *Pseudomonas aeruginosa* has become a serious concern in contemporary healthcare. The generation of carbapenemase enzymes is the primary mechanism underlying carbapenem resistance, which severely restricts treatment options and raises morbidity and mortality. For proper clinical care and efficient infection control, timely and precise detection of these enzymes is critical for optimizing antimicrobial therapy, reducing morbidity and mortality, and preventing the spread of resistant strains.

Carbapenemase production can be detected using both phenotypic and genotypic approaches, each of which has unique principles and clinical implications. Phenotypic techniques, such as the modified carbapenem inactivation method, the modified Hodge test, and the Carba NP test, identify the functional activity of resistance mechanisms and are typically affordable and appropriate for ordinary laboratory use. However, they are frequently associated with inconsistent sensitivity and specificity, as well as longer turnaround times. On the other hand, particular resistance genes like blaNDM, blaVIM, and blaIMP can be quickly and accurately identified using genotypic methods like polymerase chain reaction, multiplex PCR, and whole genome sequencing. These techniques are highly accurate but require sophisticated infrastructure and technical expertise and may not detect novel or uncharacterized resistance factors. All things considered, both methods offer useful but complementary information. While genotypic approaches provide molecular-level understanding and epidemiological significance, phenotypic approaches validate the expression of resistance. To improve infection control tactics, direct focused medication, and increase detection accuracy in healthcare settings, an integrated diagnostic strategy including both approaches is crucial.

## Introduction and background

Clinical management of healthcare-associated infections is increasingly challenged by the global rise in antimicrobial resistance, particularly among non-fermenting Gram-negative bacteria such as *Pseudomonas aeruginosa* [1]. *Pseudomonas aeruginosa* is an opportunistic pathogen responsible for a wide range of infections, including bloodstream infections, ventilator-associated pneumonia, and catheter-associated urinary tract infections. It contributes significantly to morbidity and mortality among hospitalized patients [2]. Its remarkable adaptability allows survival in diverse environments, particularly in moist hospital settings and on medical devices, making it a major cause of nosocomial outbreaks [3].

Carbapenems, including imipenem and meropenem, have historically been considered last-resort agents for the treatment of severe infections caused by *Pseudomonas aeruginosa* due to their broad-spectrum activity and strong affinity for penicillin-binding proteins [4]. However, the emergence of carbapenem-resistant *Pseudomonas aeruginosa* (CRPA) has become a serious global concern. The World Health Organization has classified CRPA as a critical priority pathogen due to its significant threat to public health and the urgent need for new therapeutic strategies.

Understanding the underlying mechanisms of carbapenem resistance is essential to appreciate the clinical significance of CRPA infections. Resistance in *Pseudomonas aeruginosa* arises from a combination of enzymatic and non-enzymatic mechanisms. Key non-enzymatic mechanisms include overexpression of chromosomal AmpC β-lactamase, loss or downregulation of the OprD outer membrane porin, and increased activity of resistance-nodulation-division (RND) efflux pumps such as MexAB-OprM. In addition, enzymatic resistance mediated by carbapenemases plays a crucial role. Among these, Ambler class B metallo-β-lactamases (MBLs), includingVIM, IMP, and NDM, are the most prevalent, encoded by genes such as blaVIM, blaIMP, and blaNDM [5].

Importantly, carbapenemase-producing *Pseudomonas aeruginosa* strains frequently harbor additional resistance determinants located on mobile genetic elements, such as plasmids, integrons, and transposons. These elements facilitate horizontal gene transfer and enable coexpression of resistance genes against multiple antibiotic classes, including fluoroquinolones and aminoglycosides. As a result, these isolates often exhibit multidrug-resistant (MDR) or extensively drug-resistant (XDR) phenotypes, significantly limiting available treatment options and complicating clinical management [6,7].

In addition to metallo-β-lactamases, certain class D carbapenemases, such as OXA-type enzymes (e.g., OXA-48), have also been reported, although less commonly in *Pseudomonas aeruginosa*. These enzymes are of concern due to their plasmid-mediated dissemination and ability to hydrolyze carbapenems, often with variable resistance profiles that may complicate laboratory detection. Their presence further highlights the complexity of carbapenem resistance and the evolving nature of resistance mechanisms across different geographic regions [5].

Given these complex resistance mechanisms, rapid and accurate differentiation between carbapenemase-producing and non-carbapenemase-producing isolates is essential, as this distinction directly impacts infection control measures and guides the selection of appropriate antimicrobial therapy, including newer agents such as ceftazidime/avibactam and ceftolozane/tazobactam [8].

Conventional diagnostic methods, although considered reference standards, are often time-consuming, with results taking up to 48-72 h. Delayed detection can lead to inappropriate therapy, which is associated with increased morbidity and mortality, particularly in critically ill patients. Therefore, the evaluation of rapid phenotypic and genotypic detection methods is crucial for timely clinical decision-making. Furthermore, in regions such as Maharashtra, understanding local epidemiological trends is essential for contextualizing resistance patterns and implementing effective infection control strategies. This review aimed to provide a comprehensive overview of current epidemiology, resistance mechanisms, and diagnostic approaches for carbapenemase detection in *Pseudomonas aeruginosa*, with a focus on their clinical relevance and emerging challenges [4,9]. This review also evaluates and compares phenotypic and genotypic methods for detecting carbapenemase-producing *Pseudomonas aeruginosa*, with an emphasis on their diagnostic accuracy, clinical applicability, and emerging challenges.

Methods

Research Strategy 

To find pertinent research on carbapenemase detection in* Pseudomonas aeruginosa*, a thorough literature search was carried out. A thorough search was conducted for articles published between 2010 and 2026 using electronic databases such as PubMed, Google Scholar, ScienceDirect, and Scopus. *Pseudomonas aeruginosa*, "carbapenem resistance," "carbapenemase detection," "phenotypic methods," "genotypic methods," "PCR," "Carba NP test," "mCIM," and "blaNDM," "blaVIM," and "blaIMP" were among the keywords and Boolean operators utilized. To improve search sensitivity, pertinent Medical Subject Headings (MeSH) phrases were also used. Only English-language peer-reviewed publications, including original studies, systematic reviews, and meta-analyses, were taken into account.

**Figure 1:**
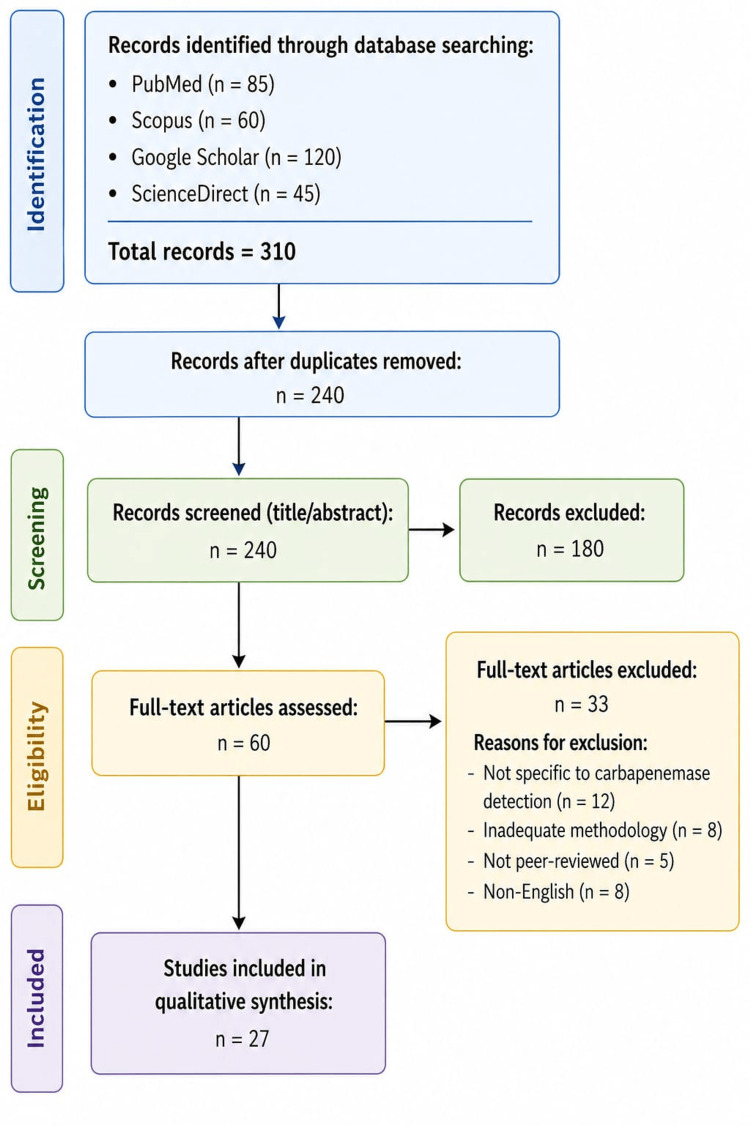
Flow diagram of study selection process.

Studies that assessed phenotypic and/or genotypic diagnostic techniques with published performance characteristics such as sensitivity, specificity, or turnaround time, and focused on carbapenem resistance in *Pseudomonas aeruginosa,* were considered. Excluded were studies that were not specific to *Pseudomonas aeruginosa*, lacked adequate methodological description, and were not peer-reviewed. The concepts, benefits, drawbacks, and practical applications of several diagnostic techniques were compared using data extraction and qualitative synthesis. To guarantee dependability and clinical relevance, more focus was placed on standardized procedures and protocols, especially those suggested by the Clinical and Laboratory Standards Institute. This review is a narrative synthesis. We did not calculate pooled effect sizes or statistical significance. Instead, findings were qualitatively synthesized based on the included studies (Figure 1).

Statistical Analysis and Data Synthesis

In this review, we synthesized the reported prevalence of carbapenem-resistant *Pseudomonas aeruginosa* (CRPA) using a random-effects meta-analysis, as several of the included studies reported comparable outcomes, such as resistance rates and patient clinical outcomes. We extracted data on resistance rates, sample sizes, and patient outcomes from each study (e.g., Pedro et al. [1], Ahmadi et al. [4], Ramatla et al. [10]). Pooled prevalence estimates were calculated, and heterogeneity was assessed using the I² statistic. We considered I² values above 50% as indicating substantial heterogeneity. For each pooled estimate, we reported 95% confidence intervals (CIs) and two-sided p-values.

## Review

Epidemiology

Due largely to variations in antimicrobial stewardship strategies and infection control practices, the global distribution of carbapenem-resistant *Pseudomonas aeruginosa *(CRPA) shows both a high overall burden and significant regional diversity [10]. Meta-analyses conducted between 2014 and 2024 estimate the global pooled prevalence of CRPA in clinical and surveillance samples to be approximately 34.7%. Europe reports the highest regional prevalence (47.6%), followed by Asia and Africa. In certain cohorts from Japan, resistance rates as high as 98.2% have been reported [10-12].

In the United States, approximately 10-30% of *Pseudomonas aeruginosa* isolates from healthcare settings exhibit carbapenem resistance, although the proportion of isolates harboring carbapenemase genes remains comparatively lower than in other regions. Despite this, surveillance data indicate a significant risk of rapid dissemination due to horizontal gene transfer. Globally, the prevalence of carbapenemase-producing CRPA continues to increase at an alarming rate [4,13].

Regional Epidemiology of CRPA

Asia: Within Asia, diverse patterns of CRPA have been observed. Notably, regional studies from Maharashtra, India, highlight the growing burden of CRPA. A retrospective study conducted in Nagpur between May 2024 and April 2025 reported *Pseudomonas aeruginosa* as the third most common Gram-negative organism, accounting for 17.05% of all culture-positive isolates. The incidence peaked in September 2024, possibly due to increased environmental humidity. Similarly, in a medical intensive care unit in Aurangabad (2023-2024), *Pseudomonas aeruginosa* accounted for 29.41% of isolates, with a carbapenem resistance rate of 44% [14,15]. These findings underscore the significant contribution of CRPA to ICU-associated morbidity in Maharashtra and align with broader regional trends across Asia.

High-Risk Clones and Transmission Dynamics

Globally recognized high-risk clones, including ST235, ST111, and ST244, are strongly associated with the dissemination of carbapenem resistance in *Pseudomonas aeruginosa*. Among these, ST235 is particularly notable due to its widespread distribution and association with multiple horizontally acquired resistance determinants, including Guiana extended-spectrum (GES) and Verona integron-encoded metallo β-lactamase (VIM) carbapenemases. Studies from Northern China have demonstrated the spread of ST244 CRPA strains across multiple hospital departments over a three-year period, indicating significant intra-hospital transmission. These clones frequently exhibit multidrug-resistant (MDR) or extensively drug-resistant (XDR) phenotypes, severely limiting treatment options to agents such as cefiderocol or colistin [16]. Table 1 summarizes regional differences in predominant carbapenemase types and key epidemiological features [10-12,16].

**Table 1:** Regional differences and diversity of carbapenemase. NDM: New Delhi metallo-β-lactamase; VIM: Verona integron-encoded metallo-β-lactamase; IMP: Imipenemase; KPC: *Klebsiella pneumoniae* carbapenemase; OXA-48: oxacillinase-48-type carbapenemase; SPM: Sao Paulo metallo-β-lactamase; CP-CRPA: carbapenemase-producing *Pseudomonas aeruginosa*; PPE: prevalence of carbapenemase-producing enzymes

Region	Predominant carbapenemase types	Key epidemiological features
Asia (South/East)	NDM, VIM, IMP	High rates of gene co-existence (e.g., NDM and VIM).
Europe	VIM, KPC, OXA-48-like	Significant north-to-south and west-to-east gradient in resistance.
Latin America	KPC, SPM, VIM	Widespread dissemination of KPC and endemic SPM-1 in Brazil.
Middle East/Africa	NDM, IMP, OXA-48	Increasing PPE reported in recent years (2022-2024).
North America	KPC, VIM, NDM	CP-CRPA remains relatively uncommon but is increasing in frequency.

Diagnostic challenges in modern microbiology laboratory: phenotypic vs. genotypic methods

The accurate detection of carbapenem resistance in *Pseudomonas aeruginosa* is essential for guiding appropriate antimicrobial therapy, implementing effective infection control strategies, and supporting epidemiological surveillance. Given the complex and multifactorial nature of resistance in this organism, no single diagnostic method is sufficient. Therefore, both phenotypic and genotypic approaches are employed to achieve comprehensive characterization [16,17].

Phenotypic Methods - Functional Detection of Resistance

Phenotypic methods are based on the direct observation of expressed resistance mechanisms, particularly the ability of bacterial isolates to grow in the presence of carbapenem antibiotics or to enzymatically inactivate these agents [17]. These methods remain the cornerstone of routine clinical microbiology due to their practicality and clinical relevance. The commonly used phenotypic methods and their key characteristics are summarized in Table 2 [16-22].

**Table 2:** Summary of commonly used phenotypic methods for carbapenemase detection in Pseudomonas aeruginosa, including their principles, advantages, and limitations. MHT: modified Hodge test; mCIM: modified carbapenem inactivation method; DDST: double disk synergy test; EDTA: ethylenediaminetetraacetic acid; MIC: minimum inhibitory concentration; KPC: *Klebsiella pneumoniae* carbapenamase; CLSI: Clinical and Laboratory Standards Institute; CDT: combined disk test; CIM: carbapenem inactivation method; LFIA: lateral flow immunoassay

Method	Principle	Key points/features
MHT	Detects carbapenemase via enhanced growth of an indicator strain.	Simple, low-cost, subjective, and prone to false positives.
Carba NP test	Detects imipenem hydrolysis via pH change.	Rapid (≤2 h), high sensitivity, and specificity.
mCIM	Measures carbapenem inactivation	CLSI recommended, easy, 18-24 h turnaround.
EDTA-CDT	EDTA inhibits metallo-β-lactamases (MBLs).	Cost-effective, but may give false positives.
DDST	Detects synergy between carbapenem and inhibitor.	Simple, variable interpretation.
E-test (gradient diffusion).	MIC reduction with inhibitors.	Quantitative, more accurate, and relatively expensive.
Boronic acid disk test	Inhibits class A carbapenemases (e.g., KPC).	Useful for KPC; not for MBLs.
Blue-Carba test	Color change due to carbapenem hydrolysis.	Rapid, easy visual interpretation.
CIM	Assesses carbapenem degradation.	Reliable, longer turnaround time.
LFIA	Antibody detection of carbapenemases.	Rapid, limited to known enzymes.
Matrix-assisted laser desorption ionization-time of flight mass spectrometry hydrolysis assay.	Detects antibiotic degradation via mass spectrometry.	Rapid, integrates with routine identification systems.

Carbapenemase Activity-Based Assays

The Carba NP test is a rapid biochemical assay that detects carbapenemase activity through the hydrolysis of imipenem, resulting in a measurable pH change indicated by a color shift. This method provides results within a short time frame and demonstrates high diagnostic accuracy for detecting carbapenemase-producing organisms [18].

The modified carbapenem inactivation method (mCIM) evaluates the ability of a test organism to inactivate a carbapenem antibiotic, which is subsequently assessed using a susceptible indicator strain. This method is widely recommended due to its reliability and simplicity, although it requires an incubation period of 18-24 h [18].

The modified Hodge test (MHT) is an earlier method used to detect carbapenemase production through enhanced growth of a susceptible strain toward a carbapenem disk. Despite its historical importance, it is now less commonly used due to issues such as false-positive results and reduced sensitivity for certain enzyme types [16].

Inhibitor-Based Phenotypic Tests

Inhibitor-based methods use specific compounds to differentiate classes of carbapenemases. For example, ethylenediaminetetraacetic acid (EDTA) is used to detect metallo-β-lactamases by inhibiting zinc-dependent enzymatic activity. While these tests are cost-effective and relatively simple to perform, their interpretation may be subjective and less specific compared to molecular techniques [19].

Routine Susceptibility Testing

Standard antimicrobial susceptibility testing (AST), performed according to established guidelines, remains a fundamental component of resistance detection [17]. These methods determine minimum inhibitory concentrations (MICs) and provide clinically relevant information regarding antibiotic efficacy. However, they do not identify the underlying resistance mechanisms.

Advantages

Phenotypic testing confirms the active expression of resistance mechanisms at levels that may lead to therapeutic failure [17]. These methods are capable of detecting resistance arising from unknown or novel genes as they assess functional expression rather than specific genetic targets [18]. Additionally, they are generally cost-effective and suitable for routine diagnostic laboratories. Standardized protocols from organizations such as CLSI enable consistent interpretation across clinical settings [19-21].

Disadvantages

Phenotypic methods are limited by longer turnaround times due to required incubation, which can delay clinical decisions [16]. Their accuracy may be affected by variations in testing conditions, leading to inconsistent results [17]. Some assays show reduced sensitivity for certain carbapenemases and may produce false-positive results, particularly with older methods [16]. Additionally, these methods cannot identify specific resistance genes and may involve subjective interpretation, requiring confirmatory molecular testing [22].

Genotypic Methods - Molecular Detection of Resistance

Genotypic approaches focus on the identification of specific genetic determinants responsible for resistance. These methods provide rapid and highly specific detection, enabling early clinical decision-making and enhanced epidemiological tracking [23]. The commonly used genotypic methods and their key features are summarized in Table 3 [22-26].

**Table 3:** Overview of genotypic methods for carbapenemase detection, highlighting their diagnostic accuracy, turnaround time, and clinical applicability. qPCR: quantitative polymerase chain reaction; LAMP: loop-mediated isothermal amplification; DNA: deoxyribonucleic acid; WGS: whole genome sequencing; NGS: next-generation sequencing; CRISPR: clustered regularly interspaced short palindromic repeats

Method	Principle	Key points/features
Conventional PCR	Amplifies specific resistance genes.	High specificity; requires prior knowledge of gene targets.
Multiplex PCR	Simultaneous amplification of multiple genes.	Time-saving; detects multiple carbapenemase genes in one run.
Real-time PCR (qPCR)	Quantitative detection using fluorescent probes.	Rapid and highly sensitive allows quantification.
LAMP	DNA amplification at constant temperature.	Rapid; no thermal cycler needed; suitable for resource-limited settings.
DNA microarray	Hybridization of DNA to multiple probes.	Detects multiple genes simultaneously; expensive and complex.
WGS	Complete genomic analysis.	Comprehensive; identifies all resistance genes; costly and requires expertise.
NGS	High-throughput DNA sequencing.	High accuracy; useful for epidemiology and surveillance.
CRISPR-based detection	Uses CRISPR-Cas systems for gene detection.	Emerging, highly specific, rapid, and sensitive.
DNA hybridization probes	Binding of labeled probes to target DNA.	Specifically used in confirmatory testing, limited multiplexing.

Polymerase Chain Reaction (PCR)-Based Methods

Conventional PCR is widely used to detect specific carbapenemase genes such as blaNDM, blaVIM, and blaIMP*.* This technique offers high specificity but is limited to known targets and cannot identify novel resistance genes [22]. Multiplex PCR allows simultaneous detection of multiple resistance genes in a single reaction, improving efficiency and reducing resource utilization. This method is particularly useful in surveillance studies assessing the distribution of resistance determinants. Real-time PCR (qPCR) provides rapid and quantitative detection of resistance genes, often yielding results within a few hours. Its high sensitivity and specificity make it valuable in clinical settings requiring prompt diagnosis [22].

Whole Genome Sequencing (WGS)

Whole genome sequencing (WGS) provides a comprehensive analysis of resistance genes, offering precise detection. However, it requires prior bacterial culture, which delays the process. Typically, WGS takes several days, from sample collection to sequencing and data interpretation, often requiring 48-72 h or more. In contrast, phenotypic methods deliver faster results, often within hours. Thus, while WGS is highly accurate, its longer turnaround time limits its practicality for urgent clinical decision-making, making timing a crucial factor when choosing diagnostic approaches [24].

Advantages

Genotypic techniques offer significant advantages, including rapid turnaround time, high sensitivity, and precise identification of resistance genes. They are particularly valuable for infection control and epidemiological studies, as they enable tracking of resistance patterns and transmission pathways [23].

Disadvantages

Genotypic methods are limited by high cost and the need for specialized equipment and trained personnel, restricting their routine use in many laboratories [24]. They can detect only known genetic targets, making them unable to identify novel or emerging resistance mechanisms [22]. Additionally, the presence of a resistance gene does not always correlate with phenotypic expression, which may lead to discrepancies in clinical interpretation [17].

Comparative Overview of Phenotypic and Genotypic Methods

Phenotypic and genotypic methods represent complementary approaches for detecting carbapenemase production in *Pseudomonas aeruginosa*, each with distinct advantages and limitations. Phenotypic methods assess the functional expression of resistance by evaluating the ability of bacterial isolates to hydrolyze carbapenem antibiotics. Common methods include the Carba NP test, modified carbapenem inactivation method (mCIM), and modified Hodge test. These techniques are highly relevant in clinical practice as they directly evaluate the biological manifestation of resistance [20,21,25]. They are generally cost-effective and suitable for resource-limited settings. However, their sensitivity and specificity may vary, and turnaround times can be longer. Older methods, such as the modified Hodge test, have demonstrated reduced sensitivity for detecting metallo-β-lactamases such as NDM [16].

Genotypic methods, on the other hand, utilize molecular techniques such as PCR and real-time PCR to detect specific carbapenemase genes, includingblaVIM, blaNDM, and blaIMP. These approaches offer rapid and highly sensitive detection, enabling early clinical decision-making and infection control. However, they require specialized infrastructure and may not detect emerging or unknown resistance genes unless sequencing-based approaches are employed [26].

Overall, both approaches are clinically significant. Phenotypic methods confirm functional resistance, whereas genotypic methods provide molecular insights and epidemiological data. Current evidence supports a combined diagnostic approach to enhance accuracy, guide appropriate antimicrobial therapy, and improve infection control strategies [27]. The comparative features of phenotypic and genotypic methods are summarized in Table 4 [20,21,25-27].

**Table 4:** Phenotypic vs. genotypic method.

Feature	Phenotypic methods	Genotypic methods
Detection basis	Enzyme activity	Gene presence
Cost	Low	High
Turnaround time	Moderate to slow	Rapid
Sensitivity/specificity	Variable	High
Technical requirement	Basic lab setup	Advanced molecular lab
Detection of novel genes	Possible (functional)	Limited (unless sequencing)
Clinical relevance	High (functional expression)	High (molecular confirmation)

## Conclusions

Carbapenem-resistant *Pseudomonas aeruginosa* remains a dynamic threat to healthcare, demanding precise detection for effective patient management. Phenotypic methods, though cost-effective and capable of confirming functional resistance, often suffer from slower turnaround and subjective interpretation. In contrast, genotypic methods offer rapid, sensitive identification of known resistance genes, but are limited by cost and inability to detect emerging mechanisms.

By integrating both approaches, healthcare systems can ensure more accurate detection, timely intervention, and improved infection control. Looking ahead, the development of rapid, affordable molecular diagnostics and robust antimicrobial stewardship will be key to limiting the spread of multidrug-resistant *Pseudomonas aeruginosa.*
